# Excision Dominates Pseudogenization During Fractionation After Whole Genome Duplication and in Gene Loss After Speciation in Plants

**DOI:** 10.3389/fgene.2020.603056

**Published:** 2020-12-18

**Authors:** Zhe Yu, Chunfang Zheng, Victor A. Albert, David Sankoff

**Affiliations:** ^1^Department of Mathematics and Statistics, University of Ottawa, Ottawa, ON, Canada; ^2^Department of Biological Sciences, University at Buffalo, Buffalo, NY, United States

**Keywords:** gene loss, fractionation, polyploidization, whole genome duplication, plant evolution, synteny, pseudogene, genomics

## Abstract

We take advantage of synteny blocks, the analytical construct enabled at the evolutionary moment of speciation or polyploidization, to follow the independent loss of duplicate genes in two sister species or the loss through fractionation of syntenic paralogs in a doubled genome. By examining how much sequence remains after a contiguous series of genes is deleted, we find that this residue remains at a constant low level independent of how many genes are lost—there are few if any relics of the missing sequence. Pseudogenes are rare or extremely transient in this context. The potential exceptions lie exclusively with a few examples of speciation, where the synteny blocks in some larger genomes tolerate degenerate sequence during genomic divergence of two species, but not after whole genome doubling in the same species where fractionation pressure eliminates virtually all non-coding sequence.

## 1. Introduction

The evolutionary process of gene loss, through DNA excision—or sequence elimination (Eckardt, 2001), pseudogenization (Jacq et al., 1977), or other mechanism, is the obverse of gene acquisition by a genome through processes such as tandem or remote duplication of individual genes, whole genome doubling (WGD), neo- and sub-functionalization and horizontal transfer. Loss serves a number of functional and structural roles, such as in the reconfiguring of regulatory or metabolic networks or in compensating for the energetic, material, and structural costs of gene complement expansion.

An longstanding biological controversy in evolutionary genomics (Byrnes et al., 2006; van Hoek and Hogeweg, 2007) involves the question of whether duplicated genes are deleted through random excision “elimination of excess DNA” namely the deletion of chromosomal segments containing one or more genes, which we have termed the “structural” mechanism, or through targeted (possibly) gene-by gene events such as regulatory epigenetic silencing and pseudogenization, which we call “functional” mechanisms. Because it is often difficult to ascertain whether a single-copy gene is the result of the deletion of a duplicate copy, and because the outcomes of the two kinds of process may appear similar, it is often difficult to discern which one is operating.

The alignment of the gene orders of homologous genes in two related genomes, or subgenomes of an (ancient) polyploid, such as that provided by the SynMap program on the CoGe platform (Lyons and Freeling, 2008; Lyons et al., 2008), is a uniquely reliable first step in the assessment of gene conservation or loss after speciation or polyploidization. The homology of pairs of genes in the chromosomal fragments “synteny blocks” making up such an alignment, is doubly confirmed, first by the common level of sequence similarity of all the gene pairs in the block, and second by the common chromosomal context, namely the common order of the homologous genes in the two fragments, represented as follows:


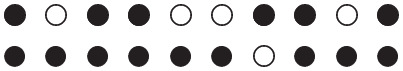

Synteny block on homeologous regions of two chromosomes.

Dark circles indicate retained genes, white circles deleted genes.

There are five retained duplicate gene pairs, four singletons on the

lower chromosome and one singleton on the upper chromosome.

In synteny blocks, it is relatively easy to see where duplicate genes have been deleted, and how many genes in a row have been lost. In this paper, we use this property of synteny blocks in devising a simple method to distinguish clearly between genomes where excision is the main mechanism for gene loss, and those where pseudogenization may also play a role.

Although the basics of polyploidy in plants have been understood for over a century (Winge, 1917), and though this process is well-attested across the entire evolutionary spectrum, from bacteria (Hansen, 1978; Tobiason and Seifert, 2006) to pre-mammalian vertebrates (Ohno, 1970), the statistical study of conservation and reduction at the genome level originates with the discovery and analysis by Wolfe and Shields of an ancient WGD in the *Saccharomyces cerevisiae* genome sequence (Wolfe and Shields, 1977). But starting with the first few plant genomes to be sequenced—*Arabidopsis, Oryza, Populus*—the realization has grown that all flowering plants species are “paleopolyploids,” re-diploidized descendants of one or more ancient polyploidization events. It is in the context of the Angiosperm/Magnoliophyte phylum or division that we have attempted to resolve the structure-function controversy (Byrnes et al., 2006; van Hoek and Hogeweg, 2007) using several modeling and statistical approaches (Zheng et al., 2009; Sankoff et al., 2010, 2015; Yu and Sankoff, 2016; Yu et al., 2020). In the present paper, however, our focus is less on how fractionated gene pairs are organized within synteny blocks, than on what happens to these genes—do they degenerate in place, or are they simply removed from the DNA sequence of the genome?

Our claim is that the overwhelming loss process is the latter: the complete excision of the gene from the genome, the elimination of the sequence of the entire gene. As such, we do not adopt any restrictive definition of a pseudogene or quantification of the various types of pseudogenes in plants, which was done in the recent definitive study of Xie et al. (2019); here we simply examine whether any DNA, and how much, remains, when a one member of a pair of homeologous genes, as identified by SynMap, is absent from a syntenic block. We will show that in the large majority of cases, there is a drastic loss of DNA, leaving only a small stretch of intergenic sequence, so that no kind of pseudogene, whatever its definition, except for very small fragments of cDNA, can be present. In other words, fractionation, and most gene loss in ancient genomes, does not tend to result in long-lasting full length or part length degenerate genes, but a relatively complete loss of the DNA. This does not mean that pseudogenes are absent or even rare in these and other genomes. Many of these may persist over many millions of years. Nevertheless, Xie et al. (2019) found that poplar has almost 25,000 pseudogenes, but <1,500 of these stem from the Salix whole genome doubling, and most of these are presumably small fragments of coding sequence.

## 2. Methods

### 2.1. Sampling of Plant Species

In each of four core eudicot plant families (or orders), we selected a pair of genomes for which annotated genome sequences are available:

1. *Populus trichocarpa* (poplar) CoGe ID 25127, and *Salix purpurea* (willow) CoGe ID 52439 in the rosid family Salicaceae,
2. *Salvia splendens* (scarlet sage) CoGe ID 55705, and *Tectona grandis* (teak) CoGe ID 55706 in the asterid family Lamiaceae,
3. *Linum usitatissimum* (flax) CoGe ID 16772 and *Hevea brasiliensis* (rubber tree) CoGe ID 16772 in the order Malpighiales, also rosids, and
4. *Malus domestica* (apple) CoGe ID 54783 and *Pyrus* × *bretschneideri* (pear) CoGe ID 37224 belonging to the same subtribe Malinae of another rosid family Rosaceae.

All these genomes have undergone at least one whole genome duplication since the ancient whole genome triplication “gamma” at the origin of the core eudicots.

### 2.2. Construction of Synteny Blocks

For each of the eight genomes individually we first carried out a self-comparison of the unmasked sequences using the SynMap program on the CoGe platform (Lyons and Freeling, 2008; Lyons et al., 2008) to construct paralogous syntenic blocks. Based on the distribution of gene pair similarities, also output by SynMap, we retained only those blocks for which the average similarity confirmed that the duplication occurred at the time of the most recent polyploidization event experienced by the genome.

For each of the four pairs of genomes, we then used SynMap to compare the two and construct orthologous synteny blocks. We again referred to the distribution of gene pair similarities in selecting only those blocks likely to have been created at the time of the speciation event at the origin of the diverging lineages leading to the two species being studied. We thus aimed to exclude synteny blocks created by polyploidization in the common ancestor of the two, including the gamma triplication, as well as blocks created in either of the two genomes by post-speciation polyploid events.

**Figure 1:**
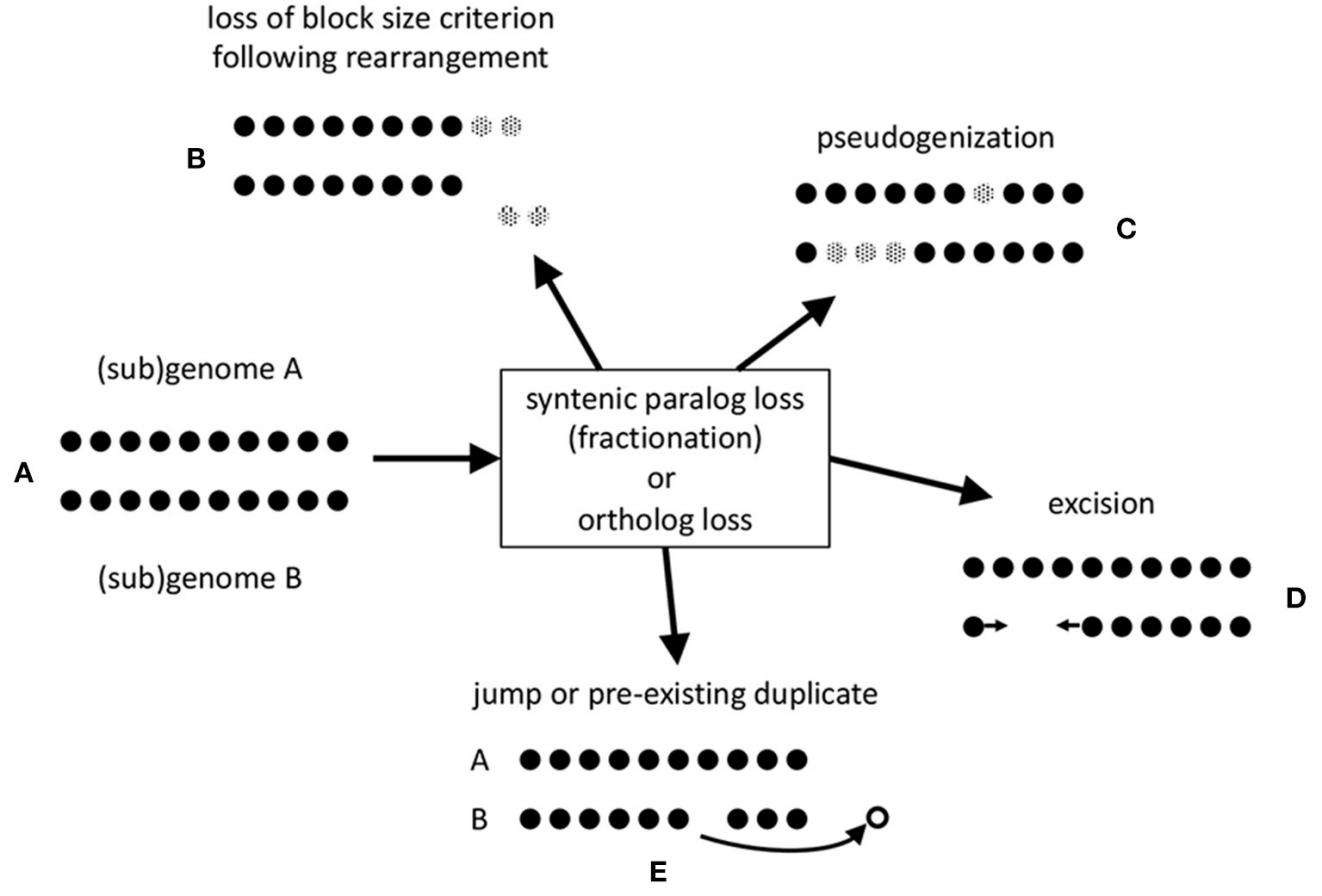
Mechanisms of loss of gene pair homology after speciation or polyploidization. **(A)** Synteny block made up of co-linear homologous pairs. **(B)** erosion of synteny block by translocation to a remote chromosomal location of a portion of sub-threshold length. **(C)** Pseudogenization. Genes rendered inoperable represented by gray dots. **(D)** Excision of DNA fragment including one or more genes. Arrows represent a new adjacency after the loss of the excised genes. **(E)** Jump of one member of pair to a different genomic location, or loss of only one of two or more homologs of the same gene.

The stringent criteria, such as a minimum number of contiguous pairs (default = 5), incorporated in SynMap tends to excludes some of the homologous gene pairs created by these genomic events (represented in Figure 1A), especially after some time has elapsed. Inversions, translocations and other chromosomal rearrangement events in a genome or in either of two related genomes, break synteny blocks into smaller pieces that may not satisfy the criteria, as illustrated in Figures 1B,E.

**Figure 2:**
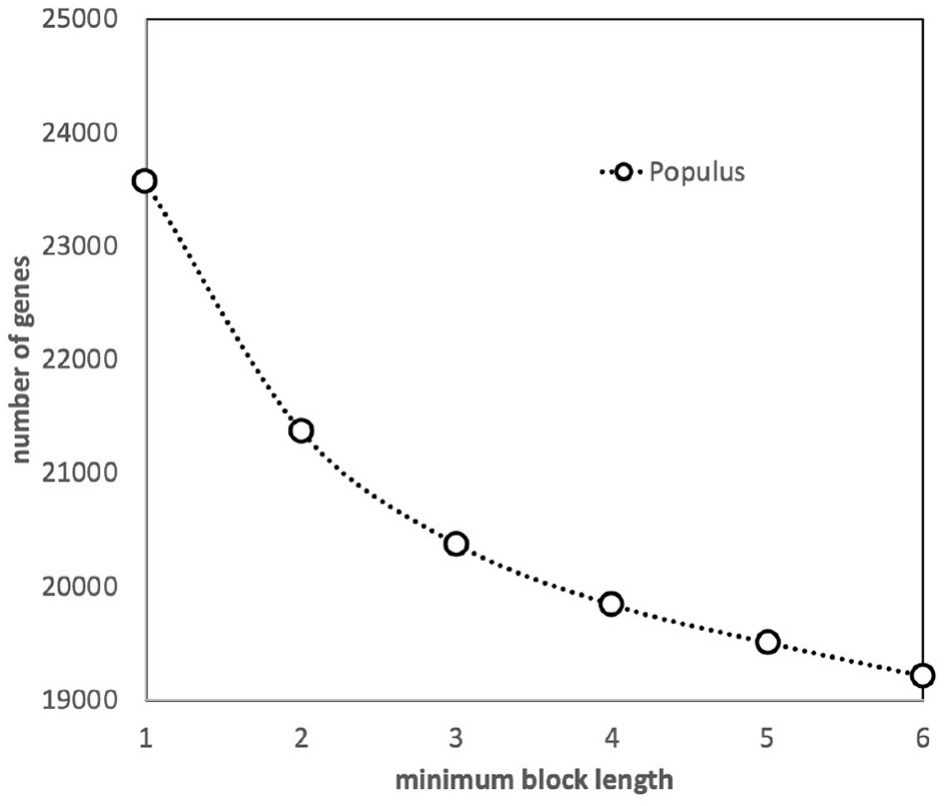
Effect of minimum block size (number of genes) on the number of genes incorporated into synteny blocks.

We have assessed the effect of the default SynMap requirement—at least five closely spaced gene pairs for a synteny block to be identified—by increasing and decreasing this threshold (see Figure 2). A slight decrease in the number of genes in blocks when the threshold is increased to 6 is simply due to the elimination of a few blocks of length 5. But as we decrease the threshold to 3, the algorithm starts to capture blocks made up of independently created but coincidentally neighboring pairs, as well as pairs where one member is already in a larger block, since a gene can be in more than one block. It becomes increasingly difficult to disentangle the behavior of duplicate gene pairs created by polyploidization from other processes of duplication and loss. Thus, we retained the default value, 5.

Since we will be focusing on pseudogenization and excision in our analysis, Figures 1C,D, we developed a method that does not favor the identification of one in favor of the other.

### 2.3. Identification of Deletion Intervals and Their Lengths

We scanned the output of the retained synteny blocks for homeologous segments on two chromosomes (or two disjoint regions of one chromosome) bounded by one or (usually) more duplicate gene pairs at both ends, where all the genes in one segment—the fractionated side—are absent, i.e., not detected by SynMap (No gene can be absent from the other segment—otherwise the ancient gene pair, if it ever existed, would not be visible.) We call the number of contiguous single-copy genes in the unfractionated side of the segment the *length* of the interval. This is the same as the number of genes that are missing from the fractionated side.

For both sides of the segment, we also determine the amount of DNA between the pairs that bound the segment. For the unfractionated side, with all the single-copy genes, this is just the size (in base pairs) of the genes plus the intergenic regions, including the initial region, after one bounding pair, and the final region, before the other bounding pair, in the segment. In the fractionated side, this includes whatever DNA remains between the two bounding pairs, which does not include any genes, according to SynMap.

Two possibilities are represented by Figures 1C,D. In the former case, pseudogenization, a gene is rendered inoperable, such as by a point mutation that creates a stop codon inside an erstwhile coding region. In the latter, a chromosomal fragment containing one or more genes is simply physically excised. To assess which of these two processes accounts for the data, we note that pseudogenization through acquiring a gene-internal stop codon, or a frameshift, leaving the gene intact, at least initially, does not shorten the length of the chromosomal region it is in. The average length of a pseudogene is roughly half of that of a functional gene (Xie et al., 2019), but this average includes the very numerous short fragments. In contrast, excision of genes, including some or all of the flanking intergenic DNA, will definitely shorten the region, leaving at most a short stretch of non-coding sequence.

### 2.4. The Visualization of Gene Density and Pseudogene Density

By plotting the average number of base-pairs in the unfractionated, or totally conserved, intervals of a given length against the length of the interval, we estimate the average size of a gene (plus the following intergenic region). In most cases we expect this plot to be approximately linear, with slope giving the average base-pairs per gene. This is just the inverse of the gene density for that interval. For the fractionated, or totally reduced, side, the number of base pairs per missing gene provides an upper limit (via its inverse) on the number of full-length pseudogenes that may be in the interval. Although most pseudogene tools were developed in the context of human or vertebrate genomes, and have limited applicability for plant genomes (Xiao et al., 2016), Xie et al. have succeeded in implementing PseudoPipe (Zhang et al., 2006) for surveying pseudogenes in a range of plant species, and their results will be seen to be consistent with ours in the analyses below.

## 3. Results

### 3.1. Willow and Poplar

**Figure 3:**
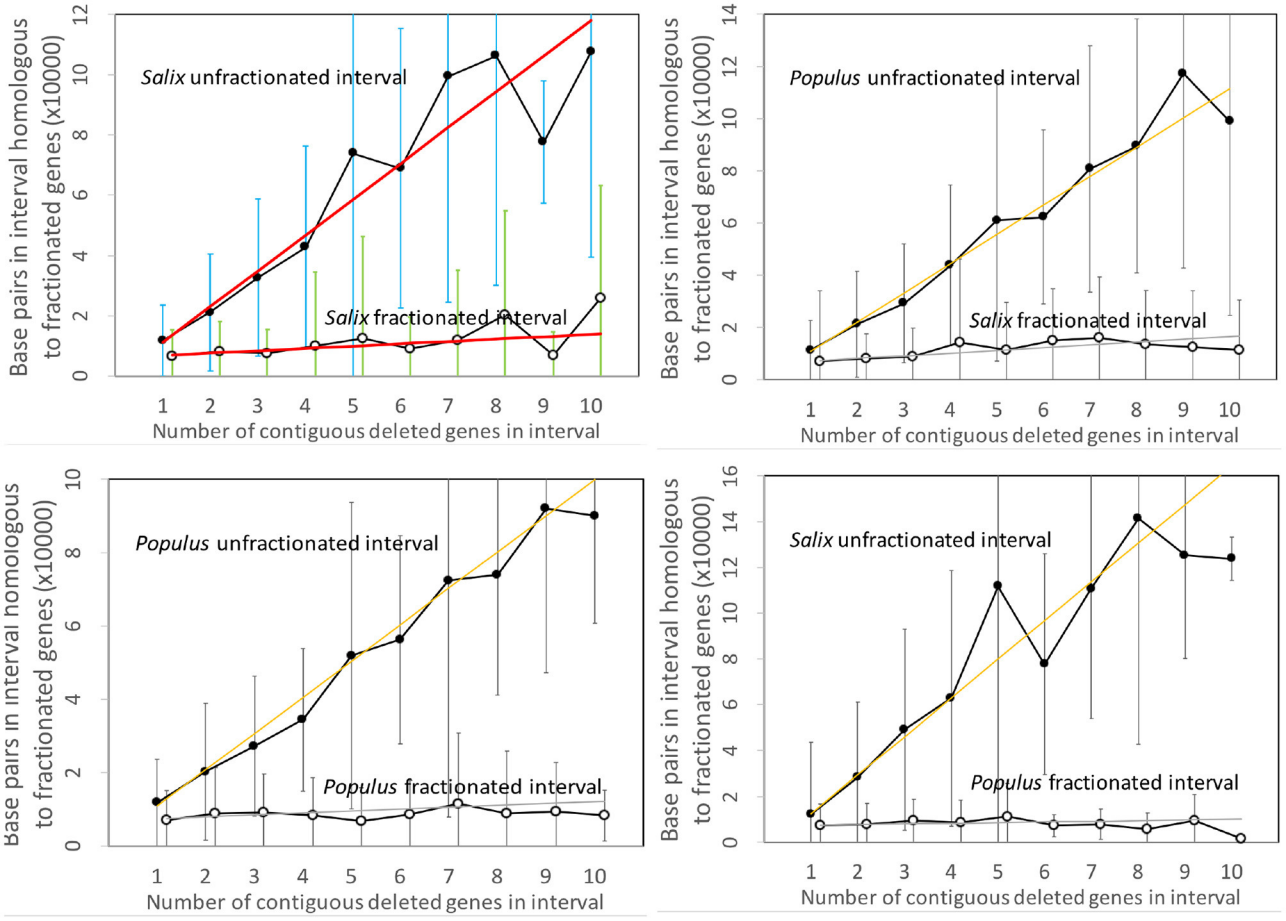
Comparison of DNA content in unfractionated and fractionated intervals in the *Salix* and *Populus* genomes. Linear regression fits are indicated. Self-comparisons on the left do not distinguish between subgenomes since these are hard to identify across chromosomes and are generally mingled due to interchromosomal rearrangements, such as reciprocal translocation and chromosome fission and fusion. The two comparisons between genomes on the right hand side analyze gene loss from each genome separately. We use the terms “fractionated” and “unfractionated” in these two panels to mean “reduced” and “conserved,” even though the polyploidization-induced fractionation does not play a role here.

Figure 3 contains the results of our analysis of the *Salix* and *Populus* genomes. The two panels on the left show the expected approximate linear growth in the number of base pairs in the unfractionated side of the interval. The great variability of the individual regions simply reflects the inhomogeneity of gene density along the length of the chromosome. In contrast, the regions in both *Salix* and *Populus* that have lost annotated genes show zero growth, with relatively little variability, as a function of the number of missing genes; they have lost almost all their DNA sequence. There cannot be significant numbers of pseudogenes, full or reduced, or other relics of the missing genes. This is striking evidence in favor of the predominance of excision.

### 3.2. *Salvia* and Teak

**Figure 4:**
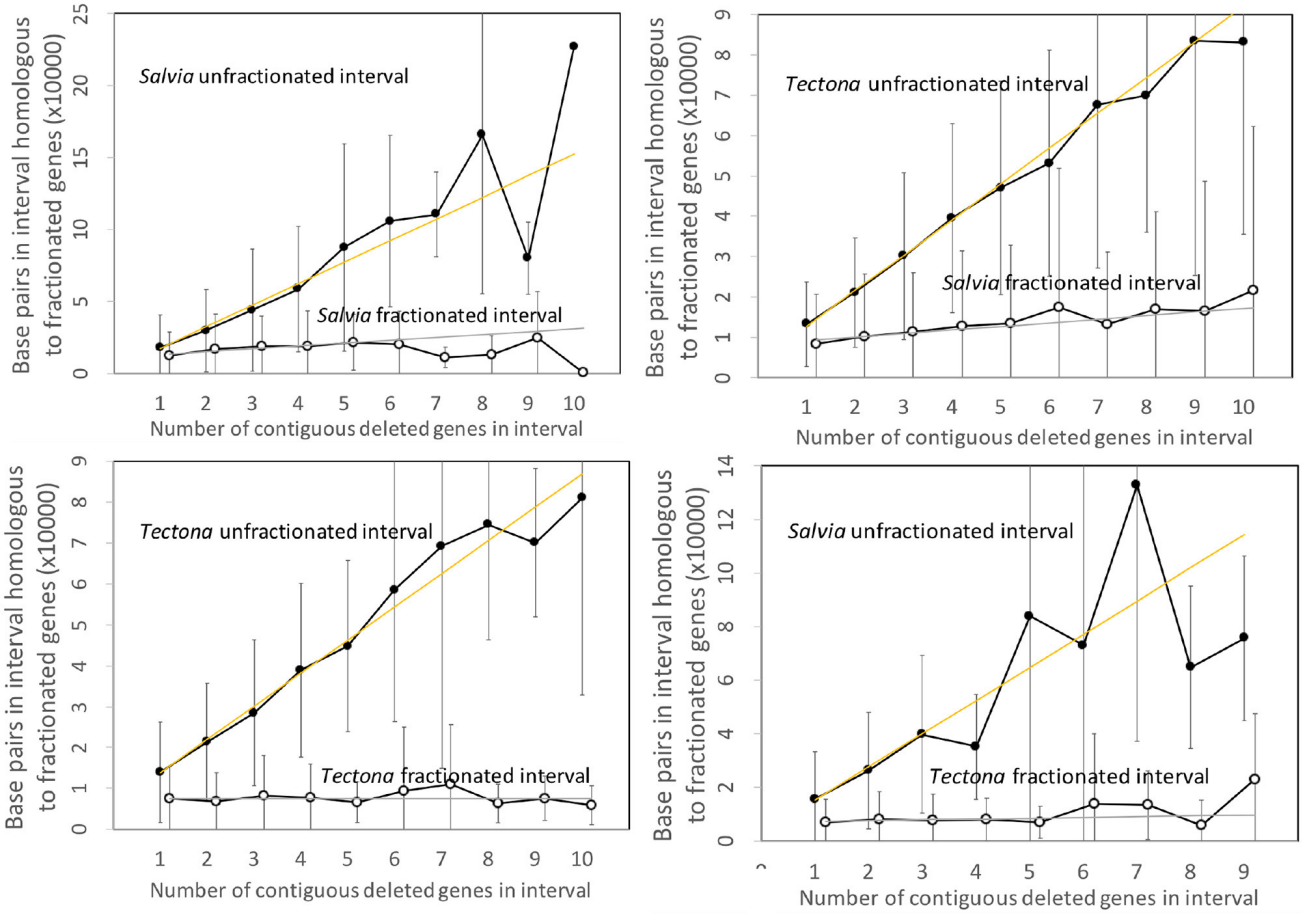
Comparison of DNA content in unfractionated and fractionated intervals in the *Salvia* and *Tectona* genomes.

Figure 4 contains the results of the corresponding analysis of the *Salvia* and *Tectona* genomes. The figures are very similar to those from the Salicacea. Some of the curves show great fluctuation of the values for the longer intervals, but this is likely due to smaller sample size. Of interest is that the DNA content of the fractionated (read: “reduced”) intervals formed after speciation show a small but steady increase, but still orders of magnitude less than the sizes of the unfractionated (“conserved”) intervals.

### 3.3. Flax and Rubber

**Figure 5:**
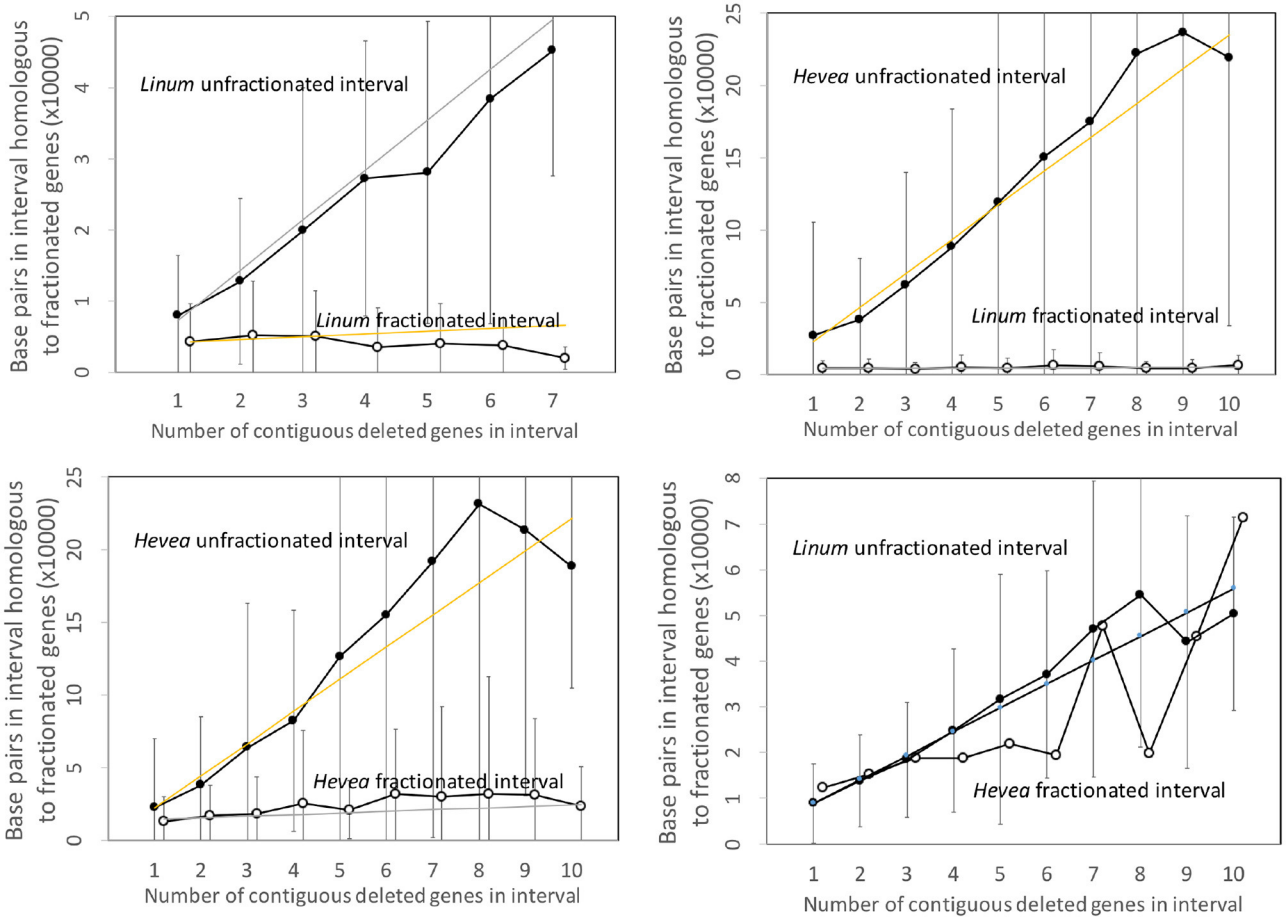
Comparison of DNA content in unfractionated and fractionated (conserved and reduced) intervals in the *Linum* and *Hevea* genomes.

Figure 5 repeats the same analysis, this time applied to the *Linum* and *Hevea* genomes. The results parallel those of the two other pairs of genomes, except for the apparently anomalous behavior of the *Hevea* intervals, where the number of base pairs attains the same level as the conserved genes in *Linum*. This, however, may be seen as an artifact of the disproportionately large genome of *Hevea* with respect to that of *Linum*. The intergenic space in *Hevea* is four or five times as great as that of *Linum*, and there is much scope for retention or acquisition of repetitive elements and other sequence over the long period since the speciation event, which occurred much earlier than the other events we study.

**Figure 6:**
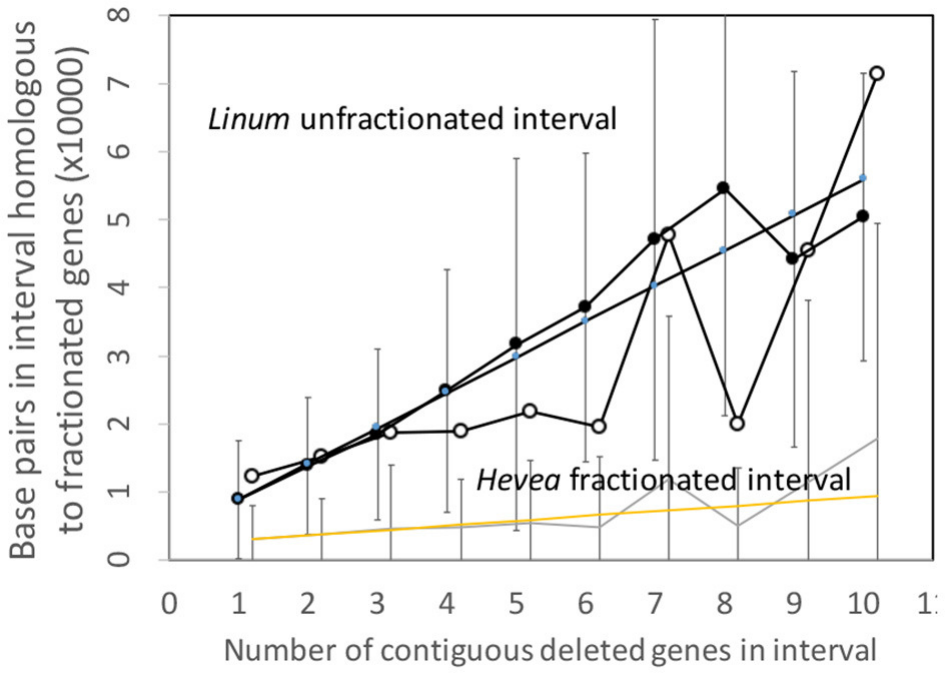
Comparison of DNA content in unfractionated and fractionated (conserved and reduced) intervals in the *Linum* and normalized *Hevea* genomes.

To put this disproportions in perspective, we can normalize the *Hevea* results by a factor which measures the difference in sizes of the two genomes. This produces the comparisons in Figure 6, which better resembles those of the Salicaceae and Lamiaceae.

### 3.4. Pear and Apple

**Figure 7:**
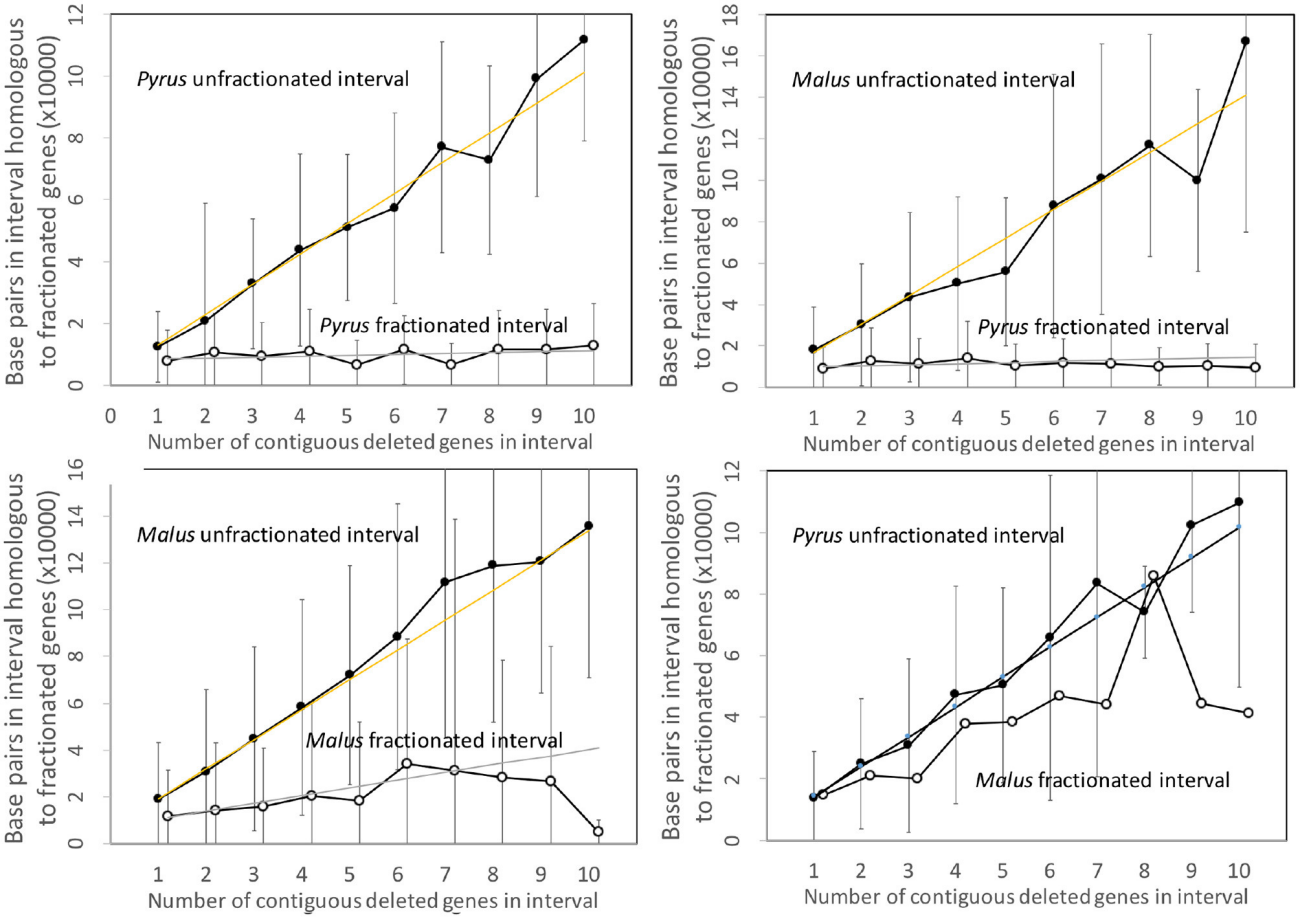
Comparison of DNA content in unfractionated and fractionated (conserved and reduced) intervals in the *Pyrus* and *Malus* genomes.

**Figure 8:**
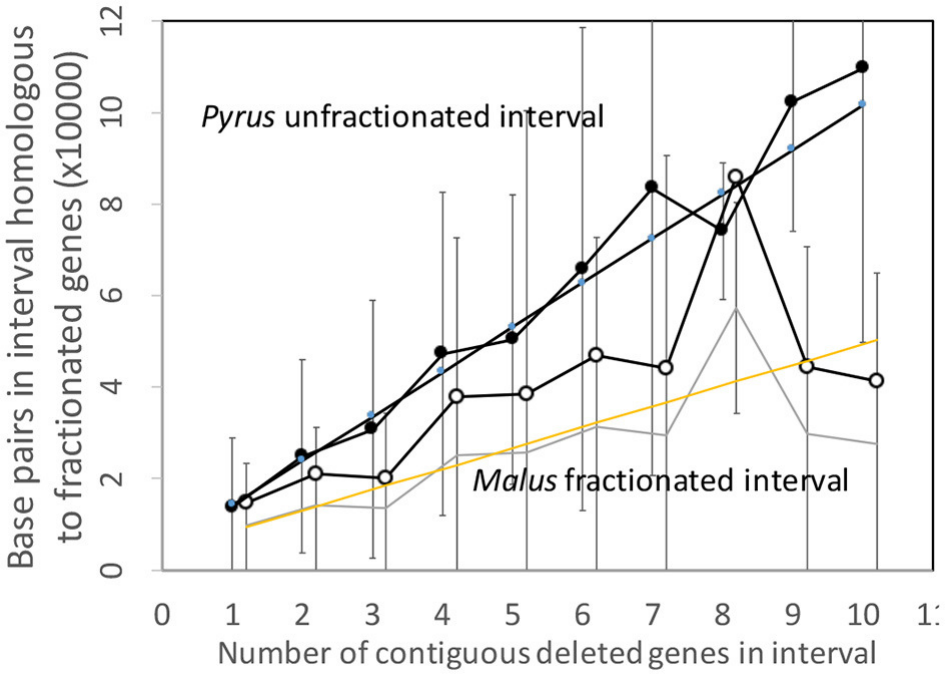
Comparison of DNA content in conserved and reduced intervals in the *Pyrus* and normalized *Malus* genomes.

Figure 7 shows the analysis of the *Pyrus* and *Malus* genomes. Here again, we have an anomalous large amount of DNA in the *Malus* reduced gene intervals after speciation. It is true that the *Malus* genome is larger than *Pyrus*, but explaining this through normalization (Figure 8) is not completely satisfactory. This is the only trend out of the thirty-two we have presented that departs from our main narrative.

### 3.5. Comparisons Across Genome Pairs

**Figure 9:**
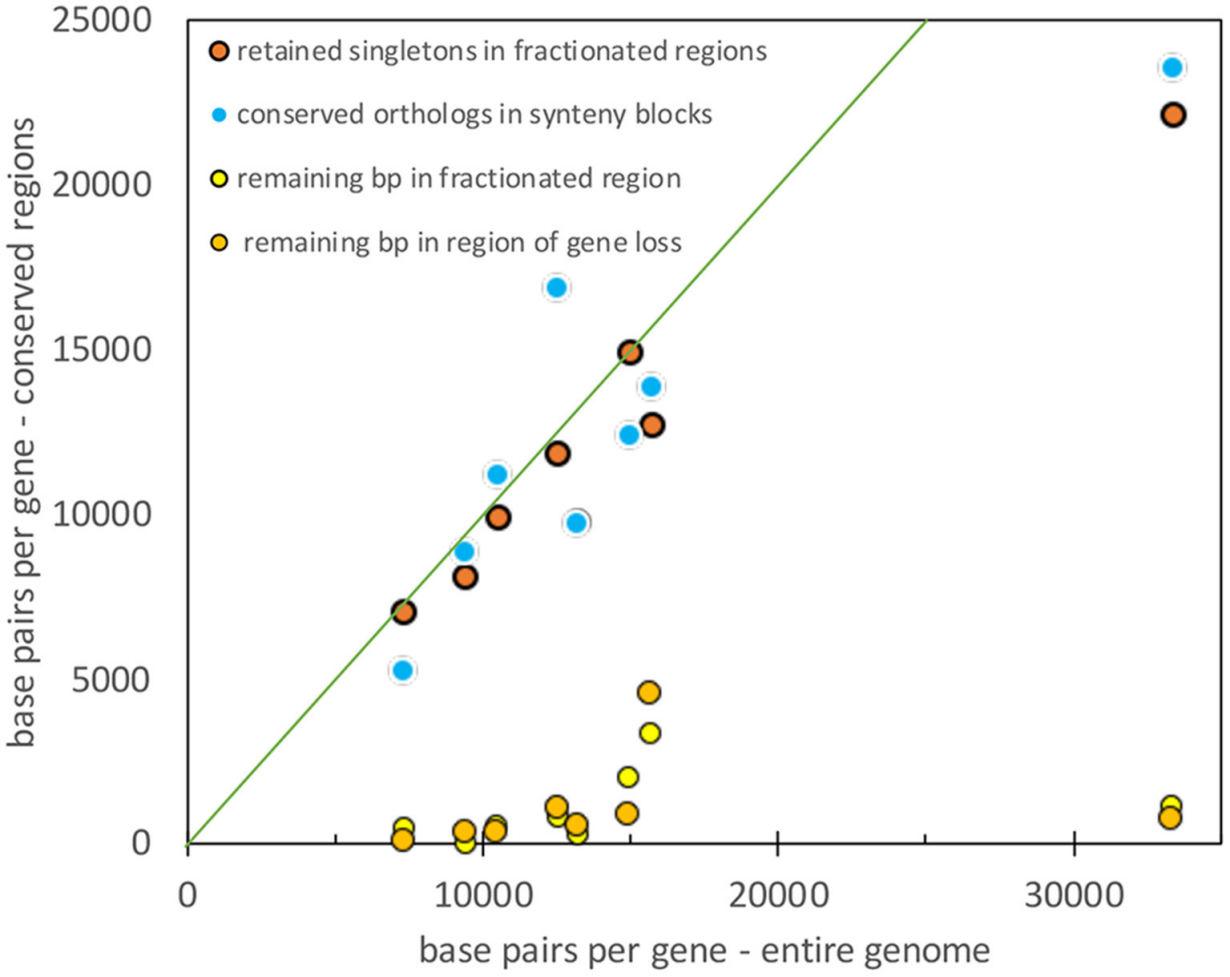
Comparison of gene density in unfractionated regions and the whole genome. Diagonal represents equality between the two densities.

To compare the results from the four pairs of genomes, we must take into account the diverse genome sizes, number of genes in a genome, and the resulting gene densities. Figure 9 shows that gene density (or rather its inverse: base pairs per length of conserved fragment) in unfractionated and conserved intervals closely tracks the average gene density (or its inverse) for the entire genome. At the same time, the residual sequence length in intervals where fractionation or gene loss has taken place is not sensitive to gene density, it remains very close to zero, as expected from an excision explanation.

We can also report, although it seems superfluous after examining Figures 3–7, 9, that a *t*-test confirms at a very high level of significance that the slopes of the two regressions in each panel are different.

### 3.6. Occurrences of Gene Translocation

To exclude other explanations of our syntenic block data, such as that in Figure 1E, we looked further into the fate of the fractionated genes in the *Populus-Salix* comparison. By setting the minimum block size to 1 in the SynMap self-comparison, we could detect all pairs of gene duplicates, not only those in synteny blocks. We then searched for pairs to the singletons identified in the original (default 5) construction of synteny blocks that we analyzed in section 3.1 above. Of the 429 out of 8,307 *Salix* singletons, we found only 429, or 5%, that were paired else where in the genome at approximately the expected similarity level. Of the 10,737 *Populus* singletons, only 742, or 7%, were paired elsewhere. Moreover, some of the pairs that were identified could have been distinct paralogs that were part of a pre-existing triplet before fractionation—such triplets or higher sets of paralogs are not uncommon. We can conclude that translocation as an alternative explanation to excision can account for only a very small fraction of the gaps in synteny blocks.

There remains the possibility that if the missing genes did not translocate out of the synteny block, the singletons may have migrated in, after the polyploidization or speciation event (Vicient and Casacuberta, 2017). The main mechanism for this would be retrotransposition. However, retroposons are generally not annotated as genes in the CoGe database, even in the unmasked genome sequences we studied, and thus would not show up as singletons. Neither are many of the singletons likely to be translocated genes: a large proportion of genes in these genomes are paired, and an equal proportion of the putatively translocated singletons would show up as pairs elsewhere in the genome in the minimum block size 1 analysis. We have already seen that this is not the case.

## 4. Conclusions

The statistical evaluation of the massive duplicate gene cohorts created by speciation or polyploidization shows that pseudogenization is either a very rare process or does not result in much stable structure. By the present time, the clear impression is that fractionation simply excises the DNA of a gene or several contiguous genes. Ongoing work to be reported elsewhere suggests that this elimination of sequence does occur piecemeal over 30 million years or even 1 million years. It is of course still possible that once a pseudogene is created, or a gene otherwise silenced, its DNA is immediately vulnerable to repeated small deletions, so that the pseudogene itself would be transient. The distinction between this and some single-event excision becomes a matter of semantics.

More surprising perhaps is that gene loss after speciation, occurring independently in two sister genomes, seems to follow the same trajectory. There is of course no genomic interaction between species pairs like *Salvia* and *Tectona*, but their common origin allows us to use one to track the gene loss pattern in the other. There remain questions of how universal excision is; in the *Salvia*-*Tectona* and *Poplar*-*Salix* comparisons it is very clear. Because of the genome size differential, it is harder to determine in *Linum*-*Hevea*, while in the case of *Malus*, though fractionation proceeds by excision, further gene loss may involve other mechanisms as well. We note that the role of differential amounts of repetitive sequence and active retroposon activity can impact this type of comparison between species, less so within one species.

Although it is difficult to say if it has any impact on our analysis, we note that speciation of apple and pear came later than their common whole genome duplication. It is the same for poplar and willow. The teak whole genome duplication occurred before speciation, but the *salvia* came after. That means that we analyzed more recent *salvia* fractionation than an earlier one that it shares with teak. The rubber-flax speciation is much more ancient than their individual whole genome duplications.

## Data Availability Statement

Publicly available datasets were analyzed in this study. This data can be found at: https://genomevolution.org/coge/.

## Author Contributions

ZY and DS planned the research, carried it out, and wrote this article. CZ developed and organized the data and participated in the planning and devising the analyses. VA contributed to the interpretation of the analyses and to understanding the pertinence of the results. All authors contributed to the article and approved the submitted version.

## Conflict of Interest

The authors declare that the research was conducted in the absence of any commercial or financial relationships that could be construed as a potential conflict of interest.
